# Positive Feedback between Transcriptional and Kinase Suppression in Nematodes with Extraordinary Longevity and Stress Resistance

**DOI:** 10.1371/journal.pgen.1000452

**Published:** 2009-04-10

**Authors:** Çagdaþ Tazearslan, Srinivas Ayyadevara, Puneet Bharill, Robert J. Shmookler Reis

**Affiliations:** 1 Department of Biochemistry and Molecular Biology, University of Arkansas for Medical Sciences, Little Rock, Arkansas, United States of America; 2 Department of Geriatrics, University of Arkansas for Medical Sciences, Little Rock, Arkansas, United States of America; 3 Central Arkansas Veterans Healthcare Service, Little Rock, Arkansas, United States of America; Huntsman Cancer Institute, United States of America

## Abstract

Insulin/IGF-1 signaling (IIS) regulates development and metabolism, and modulates aging, of *Caenorhabditis elegans*. In nematodes, as in mammals, IIS is understood to operate through a kinase-phosphorylation cascade that inactivates the DAF-16/FOXO transcription factor. Situated at the center of this pathway, phosphatidylinositol 3-kinase (PI3K) phosphorylates PIP_2_ to form PIP_3_, a phospholipid required for membrane tethering and activation of many signaling molecules. Nonsense mutants of *age-1*, the nematode gene encoding the class-I catalytic subunit of PI3K, produce only a truncated protein lacking the kinase domain, and yet confer 10-fold greater longevity on second-generation (F2) homozygotes, and comparable gains in stress resistance. Their F1 parents, like weaker *age-1* mutants, are far less robust—implying that maternally contributed trace amounts of PI3K activity or of PIP_3_ block the extreme *age-1* phenotypes. We find that F2-mutant adults have <10% of wild-type kinase activity *in vitro* and <60% of normal phosphoprotein levels *in vivo*. Inactivation of PI3K not only disrupts PIP_3_-dependent kinase signaling, but surprisingly also attenuates *transcripts* of numerous IIS components, even upstream of PI3K, and those of signaling molecules that cross-talk with IIS. The *age-1(mg44)* nonsense mutation results, in F2 adults, in changes to kinase profiles and to expression levels of multiple transcripts that distinguish this mutant from F1 *age-1* homozygotes, a weaker *age-1* mutant, or wild-type adults. Most but not all of those changes are reversed by a second mutation to *daf-16*, implicating both DAF-16/ FOXO–dependent and –independent mechanisms. RNAi, silencing genes that are downregulated in long-lived worms, improves oxidative-stress resistance of wild-type adults. It is therefore plausible that attenuation of those genes in *age-1(mg44)*-F2 adults contributes to their exceptional survival. IIS in nematodes (and presumably in other species) thus involves transcriptional as well as kinase regulation in a positive-feedback circuit, favoring either survival or reproduction. Hyperlongevity of strong *age-1(mg44)* mutants may result from their inability to reset this molecular switch to the reproductive mode.

## Author Summary

Insulin/IGF-1 signaling (IIS) impacts development, metabolism, and longevity in *Caenorhabditis elegans*. It has been viewed as a cascade of kinase reactions, chiefly phosphorylation of other kinases, leading to inactivation of the DAF-16/FOXO transcription factor. PI3K, a phosphatidylinositol kinase at the center of this pathway, converts PIP_2_ to PIP_3_, instrumental to kinase docking and activation. Here we show that PI3K deficiency elicits transcriptional inhibition of many kinases, including those of IIS itself. This creates a positive-feedback loop, wherein DAF-16/FOXO silences expression of the very kinases that would have inactivated it. In the resulting “flip-flop” genetic switch, either kinase signaling or transcriptional silencing may predominate. We discovered the transcriptional arm of this switch in infertile *age-1(mg44)* mutants, defective for PI3K activity. The absence of PIP_3_ and PIP_3_-dependent kinase activity gives free rein to gene silencing by DAF-16/FOXO. This two-tiered response could scarcely have evolved for the benefit of a sterile mutant; some components presumably serve regulatory functions in normal animals, reinforcing a switch responsive to environmental and internal signals. In *age-1(mg44)* mutants, complete inactivation of PI3K “fuses” the switch, locking worms into longevity mode. With signaling profoundly silenced, they cannot resume reproduction, but instead acquire a remarkable capacity for individual survival.

## Introduction

### IIS depends critically on the presence of PIP_3_

The IIS pathway, governing developmental arrest, metabolism and life span in *Caenorhabditis elegans* [1]–[3], is highly conserved from invertebrates to mammals. The single IIS pathway of nematodes corresponds in structure and function to two distinct pathways of mammals that signal metabolic responses to insulin, and growth response to insulin-like growth factor 1 (IGF-1), respectively [4]. IIS disruption was first discovered to enhance longevity in *C. elegans* [5]–[8], but it was subsequently shown to also extend life in *D. melanogaster* and mice [9]–[11]. Binding of insulin-like peptides to DAF-2, the insulin/IGF-1 receptor of nematodes, modulates receptor autophosphorylation and activation [12]. Active DAF-2 recruits and phosphorylates the AGE-1 catalytic subunit of phosphatidylinositol 3-kinase (PI3K), which in turn phosphorylates the regulatory subunit. Activated AGE-1 then adds a phosphate to phosphatidylinositol 4,5-diphosphate [PI(4,5)P_2_] at the inositol-ring 3-position, converting it to phosphatidylinositol 3,4,5-triphosphate [PI(3,4,5)P_3_ or PIP_3_].

PIP_3_ plays a dual role in the canonical insulin/IGF-1 pathway. The first pivotal role is membrane tethering of many signaling molecules including AKT-1 and -2, PDK-1, GSK-3 and protein kinase C [13]–[16]. PIP_3_-binding recruits or retains many kinases at the cytoplasmic surface of the cell membrane, where these enzymes and their substrates (largely other kinases) are concentrated and, by mass action, interact more efficiently. Because PIP_3_ quantitatively affects multiple components of the IIS cascade, the influence of its concentration is compounded. In addition, PIP_3_ binding to AKT-1 allosterically exposes a cryptic site recognized by PDK-1 (phosphatidylinositol-dependent kinase 1), allowing AKT phosphorylation and activation [17]. In this second role, PIP_3_ may act catalytically, in that a single molecule of PIP_3_ has the potential to bind successively to many AKT-1 molecules, enabling their activation. Although AKT-1 is the only target for which this allosteric role has been documented [17], it is possible that other signaling molecules that also possess high-affinity PIP_3_ binding sites (termed “Pleckstrin homology domains”) may be similarly controlled. In any event, we infer that insulinlike signaling should be exquisitely sensitive to PIP_3_ depletion, and that AKT-1 action (which extends far beyond IIS [18],[19]) may be absolutely dependent on the presence of at least trace amounts of PIP_3_.

The AKT-1/AKT-2/SGK-1 complex, once all of its constituent kinases have been activated by PDK-1 [20], phosphorylates the DAF-16/FOXO transcription factor at sites that block its entry into the nucleus, where it would activate or repress transcription of hundreds of target genes, including many that modulate metabolism, reproduction, life span, and resistance to oxidative stresses [21]–[24].

### IIS mutations have wide-ranging effects on longevity

Reduction-of-function mutations impairing the *C. elegans* IIS pathway (*e.g.*, *daf-2* and *age-1* mutations) cause these worms to arrest development as dauer (alternative stage-3) larvae [1]–[3]. If allowed to mature at a permissive temperature, temperature-sensitive (*ts*) *daf-2* mutant adults can attain twice the normal longevity [6]; life extension ranges from 1.1- to 2.5-fold for different *daf-2* alleles [25]. A *ts* mutant allele of *age-1*, *hx546*, was discovered by Klass [26] and reported to confer 40% and 65% life extension at 20° and 25°C respectively [5],[27],[28]. Two constitutive *age-1* alleles, *m333* and *mg44*, were initially reported to extend *C. elegans* life span by 2- to 2.6-fold [2],[8],[29]; these survivals were conducted only for first-generation (“F1”) homozygotes. We recently observed that second-generation *age-1(mg44) and (m333)* larvae slowly mature at 15–20°C into adults that live close to ten times as long as near-isogenic wild-type controls, and are highly resistant to oxidative and electrophilic stresses [30]. These exceptional worms have mean and maximal adult life spans at least three times those conferred by any other longevity-extending mutation, and throughout their adult lives they appear and behave very much like wild-type worms of a tenth their age. Addition of a second mutation in the *daf-16* gene largely or entirely reverses life-span extension and other phenotypes of all *daf-2* or *age-1* mutations examined to date [3],[6],[29],[30].

Studies of IIS-pathway mutants in *C. elegans* and other taxa have provided valuable insights into genetic mechanisms regulating life span [4]. The molecular basis for the extreme survival phenotypes of *age-1(mg44)* F2 homozygotes remains unknown, and cannot be assumed to differ only in degree from molecular mechanisms that underlie 4- to 5-fold lesser life extensions seen in other IIS mutants. The key may be PIP_3_, which plays both structural and catalytic roles in signal transduction [17],[31], and is thought to mediate both DAF-16-dependent and -independent signaling [32]. Strong *age-1* mutants, lacking all class-I PI3K activity, have no direct route to generate PIP_3_ [31]. As a result, they are expected to be deficient in all enzyme activities that require PIP_3_, either for activation by regulatory kinases, or for membrane tethering which ensures proximity of kinases to their targets [17],[31].

In the present study, we sought evidence to support such a broad role of PIP_3_ in the unique properties of *age-1(mg44)*-F2 adults. This role is an inferred one, since even normal PIP_3_ levels (in unstressed N2 worms) are too low for detection by existing methods; detectable levels are attained in starved, peroxide-stressed wild-type worms but not in similarly stressed *age-1*-mutants [33]. We were able to document the expected widespread disruption of protein kinase activity in *age-1(mg44)*-F2 worms, while making the unexpected observation that the same kinases are chiefly inhibited at the transcriptional level. Direct measurement of transcripts confirms silencing of kinase gene expression, leading us to propose a novel “hybrid” positive-feedback loop in which the IIS kinase cascade that inhibits the DAF-16/FOXO transcription factor, is itself attenuated by DAF-16-mediated transcriptional silencing of upstream kinases.

## Results

### Very long-lived *age-1* mutant worms are broadly deficient in protein kinase activity

**Figure 1:**
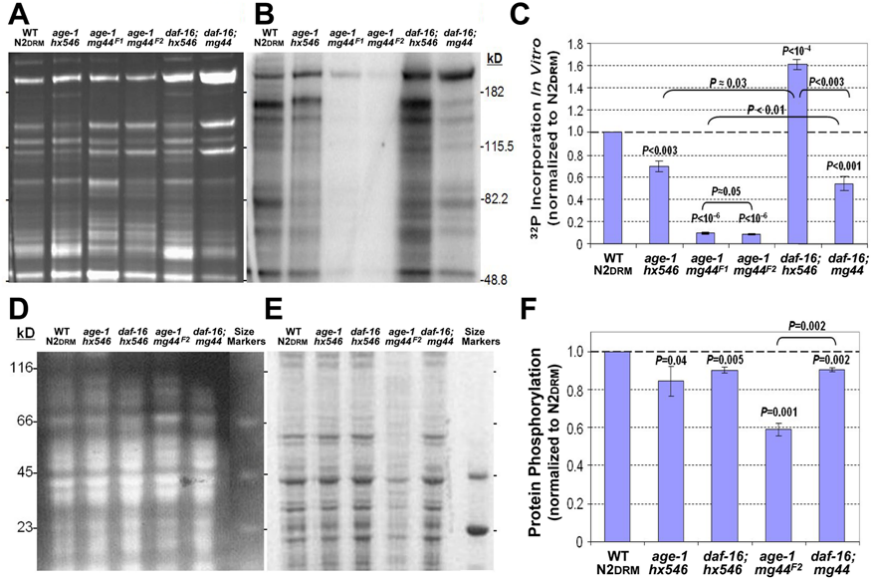
Both *in vitro* kinase activity for endogenous substrates and total phosphoprotein levels are diminished in *age-1(mg44)* homozygotes. (A–C) *In vitro* kinase activity of post-gravid or sterile adult worms, 6 days after the L4/adult molt. Kinase activity of cleared, sonicated lysates was assessed as γ-^32^P-ATP incorporation per 20-µg protein sample, in 1 min at 30°C. Samples, quenched on ice, were electrophoresed on 10% polyacrylamide-SDS gels (Invitrogen). (A) Gels stained with SYPRO Ruby (Invitrogen) to confirm constant protein loads. (B) Image of ^32^P β-emissions (Molecular Dynamics Storm) from the gel in A, dried under vacuum. (C) Data summary from 2–3 independent expansions each of N2DRM, *age-1(hx546)*, *age-1(mg44)* F1 homozygotes (labeled *age-1 mg44^F1^* *)*, *age-1(mg44)* F2 homozygotes (labeled *age-1 mg44^F2^*), and *daf-16(m26)* double mutants with each *age-1* allele. These quantitations covered the full length of each lane, not all of which is shown in A and B. (D–F) Phosphoproteins, resolved by polyacrylamide gel electrophoresis and visualized by Pro-Q Diamond staining. (D) Image of total protein stained with Coomassie blue. (E) Image of fluorescence after Pro-Q Diamond staining; note that only the two *phospho*protein markers were detected. (F) Summary of data from 3 independent expansions of each strain at adult day 6. Significance: (C, F), *P* values are shown for 2-tailed *t*-tests (unequal variance) comparing each *age-1* strain to N2DRM (values directly over bars) or comparing two strains connected by brackets (values over brackets).

The *age-1(mg44)* kinase-null mutants should be deficient in phosphatidylinositol 3,4,5-triphosphate production. Given the importance of the PIP_3_ molecule in signal transduction events originating from many membrane-receptor kinases, we anticipated that phosphorylation of numerous proteins may be impaired in those mutants. To initially assess the breadth of this impairment, we compared *in vitro* kinase activities with respect to endogenous substrates for five *age-1* mutant strains, each normalized to a wild-type N2DRM stock (Figure 1A–1C).

Panels A and B illustrate a typical experiment, and panel C summarizes results for replicate experiments with independent expansions of each group. The first-discovered and most widely used *age-1* allele, *hx546* [5],[27],[28], showed 32% less kinase activity than N2DRM (Figure 1C). However, worms bearing the *age-1(mg44)* allele had less than 10% of wild-type kinase activity, whether maternally protected first-generation (F1) or very long-lived second-generation (F2) homozygotes. The F2 worms had somewhat lower kinase activity than F1 (7.3 *vs.* 8.6% of N2DRM, *P* = 0.05), although the difference was consistently much greater for specific bands (see Figure 1B). Staining of total protein showed similar loads for all samples, although banding patterns differed (Figure 1A). One obvious difference between *age-1(mg44)* and the other strains is that these mutants are totally infertile in the F2 generation, despite the presence of syncytial nuclei [30]. Several controls exclude this as an explanation for the mutants' lack of kinase activity. *age-1(mg44)* F1's have similar kinase levels when gravid (day 2 of adulthood) or post-gravid (adult day 6; see Figure S1). Moreover, N2DRM eggs contain about half as much kinase activity as their parents, per weight of protein (Figure S1), so their *absence* would not reduce kinase activity in any case. Deficiency of kinase activity is not a characteristic of dauer larvae, which exhibit a level comparable to that of N2DRM adults (Figure S1).

The consequences of adding a *daf-16* mutation are quite different for the two *age-1* alleles: in *age-1(hx546)* worms, the *daf-16(m26)* mutation more than doubled the *in vitro* kinase activity, from 68% of wild-type to 160%, whereas this mutation restored less than half of the kinase deficiency due to the *age-1(mg44)* allele. Insofar as kinase suppression is reversed in *daf-16; age-1(mg44)* double mutants, we infer that activity is inhibited in part through the DAF-16/ FOXO transcription factor. However, reversion is far from complete, by either the *m26* (point-mutant) or the *mu86* (large-deletion) allele of *daf-16* (see Figure 1C and Figure S1). This implies that a large proportion of observed kinase silencing is DAF-16-independent—perhaps reflecting direct effects of PIP_3_ depletion on kinases other than AKT, or AKT targets other than DAF-16/FOXO.

To corroborate low protein-kinase activity of *age-1(mg44)* adults, and to distinguish whether they are deficient for many protein kinases or a few very active ones, we constructed arrays of 70 synthetic peptides comprising 50 near-consensus kinase sites from the *C. elegans* proteome and 20 from mouse or human proteins. Phosphorylation *in vitro* was observed on 29 peptides, representing potential substrates for at least 18 distinct kinases (Figure S2 and Table S1). Protein kinase activity in extracts from *age-1(mg44)* F2 adults was reduced by 1.8- to >8-fold, relative to isogenic N2DRM postgravid worms (each at nominal *P*<0.05), for 22 of the 29 kinase targets that were phosphorylated *in vitro*. Addition of the *daf-16(mu86)* mutation produced essentially complete reversion, or hyper-reversion (activity>N2DRM), for 17 of those 22 peptides.

**Figure 2:**
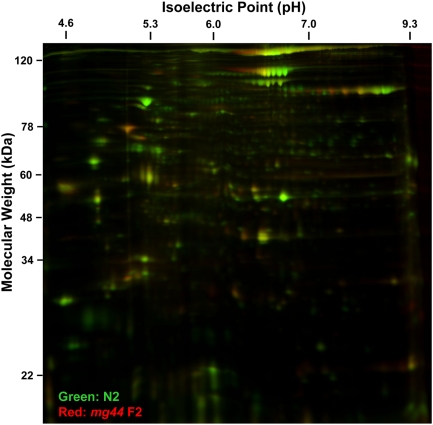
Most phosphoproteins are depleted in *age-1(mg44)* F2 adult worms. A 2-dimensional polyacrylamide gel electrophoresis resolves dual-labeled phosphoproteins, enriched on phospho-affinity columns (Qiagen), from *age-1(mg44)* F2 worms collected 10 d after the L4/adult molt (red fluorescence) and from post-gravid, near-isogenic N2DRM controls 6 d after becoming adults (green fluorescence). A total of 1,669 spots were identified and quantified for each fluor after co-electrophoresis. Red/green ratios equal to or greater than 2 (*i.e.*, at least 2-fold more abundant in *age-1(mg44)* than in N2DRM adults) were observed for 13 spots; 30 had ratios of 1.2–2.0; 122 were essentially constant (ratios of 0.8–1.2); 305 fell between 0.5 and 0.8; and 1,199 were reduced at least twofold in abundance in *age-1(mg44)* adults. A reduction of at least 3-fold was seen for 784 spots, and ≥5-fold for 287 spots.

In view of the reduced protein-kinase activity of *age-1(mg44)* worms, we anticipated that their steady-state level of protein phosphorylation would also be depressed. To assess this, phosphoproteins were separated by acrylamide gel electrophoresis and compared among wild-type and *age-1*-mutant strains of *C. elegans* (Figure 1D–1F). Total protein staining (panel D) demonstrated even loading, while panel E shows the same gel stained with Pro-Q Diamond to detect and quantify phosphoproteins. Results for three replicates (independent expansions of each strain) are summarized in panel F. Relative to wild-type N2DRM, *age-1(hx546)* worms had 16% less phosphoprotein staining (marginally significant at *P*<0.05), while *age-1(mg44)* homozygous F2 adults showed a 41% reduction in steady-state phosphoprotein level (*P*<0.001). The *daf-16(m26)* mutation restores either allele to ∼92% of the N2DRM level. This finding is also supported by 2-D dual-fluor phosphoprotein gels (Figure 2), in which 72% of the phosphoprotein spots resolved (1199/1669) were reduced at least twofold in F2 *age-1(mg44)* adults relative to N2DRM. The deficiency of total phosphoprotein content is less pronounced than that of protein kinase activity, in *age-1(mg44)*-homozygous F2 adults, which is not surprising given that phosphoprotein levels reflect the steady state, *i.e.*, a balance between kinase and phosphatase activities. We present evidence (next section) that the PTEN phosphatase is indeed downregulated in *age-1(mg44)*.

### Transcriptional suppression of signal-transduction genes in *age-1(mg44)* worms

F2 homozygotes for *age-1(mg44)* are expected to produce only truncated class-I PI3K_CS_, lacking the kinase domain and C-terminus of the protein. These worms indeed lack the main bands recognized by antibodies to the AGE-1 C-terminal region (Figure S3); residual bands may represent class-II and -III homologs of AGE-1. PIP_3_, formed exclusively by class-I PI3K, should thus be greatly reduced or absent. PIP_3_ is strictly required for PDK-1 activation of AKT kinase, which then phosphorylates and inactivates DAF-16/FOXO. Kinases that require PIP_3_ binding for membrane tethering or kinase activation [17],[31], such as AKT, PDK-1, and SGK-1, are expected to show marked suppression of activity, which cannot be directly reverted by a *daf-16* mutation. The surprising observation that mutations to *daf-16* restore nearly half of the *age-1 (mg44)*-F2 kinase deficiency, and >70% of its phosphoprotein deficit, implies that their inhibition must be mediated in part by DAF-16/FOXO. Such regulation could be direct (DAF-16 suppresses transcription of many kinase genes) or indirect (DAF-16 suppresses one or a few kinases, or stimulates one or a few phosphatases, which then suppress other kinases by impeding or opposing their phosphorylation). To test *direct* effects of DAF-16/FOXO, we used real-time polymerase chain reaction (RT-PCR) to quantify the effects of *age-1* alleles, with or without added inactivation of *daf-16*, on transcript levels for IIS genes and a panel of other signaling components, representing a wide range of transduction pathways.

#### IIS genes

**Figure 3:**
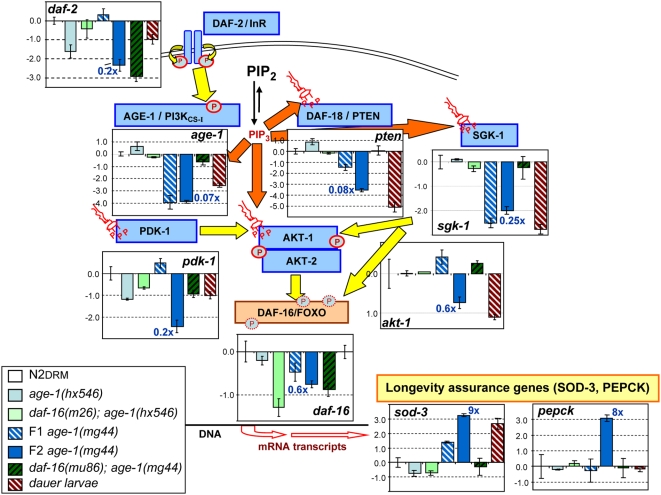
Transcriptional suppression of IIS genes in *age-1(mg44)* F2 adults. Transcript levels were assayed by real-time polymerase chain reaction (RT-PCR). Expression histograms are shown superimposed on a simplified schematic of the IIS pathway. Yellow arrows indicate protein phosphorylations (symbolized by circled P's), and orange arrows indicate binding/activation by phosphatidylinositol 3,4,5-triphosphate (PIP_3_, red “structural” symbols). Transcriptional outputs are indicated by open block arrows. Within each histogram, means±SEM are shown on a log(2) scale for steady-state transcript level, comparing wild-type to four *age-1* mutant populations and to dauer larvae. For each strain indicated, fold changes are shown (*e.g.*, “3×”), of *age-1(mg44)*-F2 relative to N2DRM. The a*ge-1(mg44)* worms are post-gravid F1 homozygotes at days 8–9 of adulthood, or F2 homozygotes at day 10 after the L4/adult molt; other strains (N2DRM, *age-1(hx546)*, and *daf-16(mu86); age-1(mg44)* double mutants) were harvested as post-gravid adults (day 6 of adulthood), or as dauer larvae (N2DRM only, from starved, dense cultures 1 day after >98% of worms had become resistant to 1% sodium dodecyl sulfate). Transcript levels were assayed for 3–8 independent biological replicates, with two cDNA syntheses and two RT–PCRs for each. All results were normalized to the mean of three control genes (β-actin, T08G5.3, and Y71D11.3) that did not differ significantly among strains. Significant differences, relative to N2DRM controls, are indicated by asterisks in Table 1.

We first assessed genes involved in insulin-like signaling. Results of a comparison of independent biological replicates for each strain are shown graphically in Figure 3, and data for these and additional genes are tabulated (including statistical significance) in Table 1. Transcript levels in *age-1(mg44)* F2 adults were found to be markedly reduced, by factors of 4- to 14-fold, for all kinase genes of the IIS pathway except *akt-1* and *akt-2*. This silencing extends even to the *age-1* gene itself (encoding the class-I catalytic subunit of PI3K) and, to a lesser extent, the class-II and class-III PI3K_CS_ genes (Table 1). Three genes encoding transcriptional cofactors of DAF-16/FOXO were significantly inhibited in *age-1(mg44)*: *smk-1* [34] and *sir-2.1* [35], encoding coactivators that reinforce unphosphorylated DAF-16/FOXO, and also *par-5*, encoding a member of the “14-3-3” family that binds and inactivates IIS-phosphorylated DAF-16/FOXO [36]. In addition, the *daf-18* gene encoding PTEN phosphatase (opposing PI3K) [37],[38] was attenuated >12-fold.

**Table 1:** Expression of signal-transduction genes in *C. elegans* strains, assessed by real-time polymerase chain reaction.

Pathway	Gene	Protein Function / Notes	DAF-16 sites†	*hx546*	*daf-16; hx546*	F1*mg44*	F2 *mg44*	*daf-16; mg44*	dauers
**Insulin/IGF-1 Signaling (IIS) pathway**									
	***ins-1***	DAF-2/IIS antagonist	1, 1	16.0*	4.0	−	10.0***	**3.5°**	80.0****
	***ins-5***	neuronal expression; no effect of RNAi	1, 3	1.9	0.90	24.0**	4.5*	1.0	7.4*
	***ins-6***	poss. agonist; no effect of RNAi	0, 2	1.9	1.4	10.7**	3.2*	1.2	2.2
	***ins-7***	DAF-2/IIS agonist; RNAi extends life	0, 2	5.3*	5.3*	0.95	**0.55°**	1.2	1.6
	***ins-14***	possible DAF-2 / IIS agonist	0. 0	1.5	1.1	26.7*	6.8****	1.8*	22.0*****
	***ins-18***	IIS antagonist; structure similar to INS-1	1, 1	1.4	1.5	**3.0°**	5.0***	1.1	16.1****
	***daf-2***	insulin/IGF-1 receptor	1, 2	**0.38°**	0.90	**1.6°**	0.20*****	0.19*****	**0.55°**
	***ist-1***	insulin-receptor substrate 1 (IRS-1)	3, 0	2.0	0.55	**1.3°**	1.8*	0.90	**6.5**
	***age-1***	PI3Kcs (class-I)	0, 0	1.6	0.80	0.06***	0.07*****	0.63	0.17**
	**F39B1.1**	PI3Kcs (class-II)	−	0.90	0.80	0.90	0.25***	0.90	0.90
	***vps-34***	PI3Kcs (class-III); vesicular trafficking	0, 2	1.7	0.90	0.90	0.37**	1.0	0.44
	***daf-18/pten***	PIP_3_ phosphatase (opposes AGE-1)	0, 0	1.8	1.0	**0.35°**	0.08*****	1.0	0.03*****
	***sgk-1***	serum/glucocorticoid-dependent kinase 1	−	1.0	0.80	0.18*	0.25**	0.85	0.15*
	***pdk-1***	phosphoinositide-dependent kinase 1	2, 1	0.44	0.63	1.4	0.18**	**0.52°**	**0.50°**
	***akt-1***	ortholog of S/T kinase AKT/PKB (RAC-α)	3, 1	1.0	1.0	1.3	0.60	1.2	**0.46°**
	***akt-2***	homolog of S/T kinase AKT/PKB (RAC-γ)	−	1.90	0.64	**5.2°**	**2.6°**	0.50	3.2
	***daf-16***	FOXO1 / FOXO3 forkhead transcr'n factor	3, 3	0.90	0.40	0.70	**0.60°**	**0.50°**	1.0
	***sod-3***	Mn-superoxide dismutase (mitoch. SOD)	−	0.57	0.55	**3.0°**	9.0***	0.90	7.0*
	***pepck***	phosphoenolpyruvate carboxykinase	0, 1	0.90	1.1	0.80	8.5*	0.95	0.90
**DAF-16-interacting transcriptional regulators**									
	***cst-1***	DAF-16 kinase, responds to oxidat.-stress	0, 0	2.3	0.90	1.3	1.0	1.2	**2.6°**
	***bar-1***	β-catenin transcript'n factor (Wnt pathway)	0, 3	0.60	1.1	−	0.60	0.70	−
	***smk-1***	transcr'l coactivator of DAF-16 and PHA-4	1, 3	0.72	1.1	0.70	0.28**	0.87	0.18***
	***par-5***	14-3-3 family; sequesters pDAF-16	1, 3	1.5	1.1	0.48	0.28**	1.1	0.13***
	***ftt-2***	14-3-3 family; sequesters pDAF-16	−	1.5	0.90	0.62	0.86	1.0	0.63
	***sir-2.1***	transcriptional coactivator of DAF-16	0, 1	0.91	1.1	0.95	0.26**	0.89	0.29**
**TGF-β signaling**									
	***daf-7***	TGF-β family member (ligand, agonist)	−	7.0*	2.6	3.0	5.3**	1.2	19.7**
	***daf-1***	TGF-β family receptor type I	1, 0	**2.2°**	**1.5°**	**0.50°**	0.22*****	1.6	1.1
	***daf-4***	TGF-β family receptor type II	1, 3	1.5	1.0	0.60	0.24***	1.5	1.2
	***daf-12***	VDR homolog, controlled by TGF-β, IIS	−	1.6	1.0	0.70	1.6	0.80	**3.1°**
	***daf-3***	SMAD transcription factor	2, 1	**1.5°**	0.90	0.30***	0.30**	1.9**	1.7*
		**▸ ** ***TGF-beta signaling outputs to p38 and ERK MAPK pathways***							
**AMPK/TOR pathway (nutrient sensing)**									
	***aak-1***	AMP-dependent kinase 1	2, 1	1.2	0.80	0.47	0.17***	1.0	0.05*
	***aak-2***	AMP-dep't. kinase 2 (activates DAF-16)	−	**1.7°**	1.5*	0.60**	2.2**	0.57	6.4*
	***let-363***	FRAP/mTOR ortholog; part of PIK family	1, 0	0.70	0.70	1.0	0.25**	0.60	0.47
	***daf-15***	ortholog of RAPTOR, mTOR reg. subunit	2, 0	0.80	0.90	0.80	0.15***	0.80	0.22**
	***rsks-1***	S6K; ribosomal S6 protein kinase 1	1, 0	1.6	0.90	1.0	1.5	1.2	3.9*
**p38-MAPK (stress response, immune response)**									
	***pmk-1***	p38 MAPK 1 (mitogen activated prot. kinase)	3, 0	−	1.0	**3.8°**	0.60	0.70	1.0
	***pmk-2***	p38 MAPK 2 (mitogen activated prot. kinase)	2, 0	−	0.20*	0.40	0.20*	0.18*	0.50
	***pmk-3***	p38 MAPK 3 (mitogen activated prot. kinase)	2, 0	−	0.40	1.2	0.56	0.20*	1.5
	***atf-2***	cAMP-dep. transcription factor family (+)	0, 1	2.5	1.1	44.1***	3.9*	**2.6°**	9.6**
		***▸ p38 MAPK signaling also activates DAF-3***							
**JNK-MAPK (immune signaling, stress response)**									
	***jnk-1***	Jun N-terminal kinase 1	0, 0	1.2	1.4	0.95	3.5***	1.2	13.2*****
	***jun-1***	JUN subunit, AP-1 transcription factor (+)	2, 2	0.74	1.0	1.1	**0.6°**	0.95	2.2*
		***▸ JNK signaling also converges on DAF-3 and DAF-16/FOXO***							
**ERK-MAPK (growth-factor and immune responses)**									
	***let-60 RAS***	small membrane GTPase co-receptor	0, 0	0.50	0.30	0.18*	0.16***	0.50	0.30
	***lin-45***	Ser/Thr kinase of MEK-2, activ by LET-60	0, 1	1.0	0.80	1.1	0.20**	0.90	0.33**
	***mek-1***	stress-response ERK for JNK-1, MPK-1	3, 1	0.75	0.50	0.30*	0.23*****	**0.38°**	0.07****
	***mpk-1***	MAPK, transduces develop'l RAS signals	0, 2	0.80	1.8	2.1	0.23*	1.0	0.21*
	***gsk-3***	glycogen-synthase kinase 3	0, 0	0.56	0.50	**0.28°**	0.20**	0.90	0.13*
	***skn-1***	oxid.-damage-response TF, inhib'd by IIS	0, 0	1.3	1.0	0.55	0.30*	1.6	0.60

**Key:** −, not assessed. **Significance of difference from N2DRM (by 2-tailed** ***t*** **-test):** °, nominally significant at *P*<0.05.

*, *P*<0.01; **, P<0.001; ***, P<1E−4; ****, P<1E−5, *****, P<1E−6.

**†:** Number of exact consensus DAF-16 sites in upstream 5-kb span, (a, b), where **a** = GTAAA(C/A)AA, and **b** = CTTATCA.

For each gene tested, wild-type N2DRM adults are compared to four *age-1* mutant populations and to dauer larvae. The a*ge-1(mg44)* worms are F1 homozygotes at days 8–9 of adulthood, by which time they were post-gravid, or F2 homozygotes at day 10 (18 days post-hatch); other groups (N2DRM, *age-1(hx546)*, and *daf-16(mu86)*; *age-1(mg44)* double mutants) were harvested when post-gravid (days 6–8 of adulthood), or as dauer larvae (N2DRM only) from starved, dense cultures 1 day after >98% of worms had become resistant to lysis by 1% sodium dodecyl sulfate. Transcript levels were assayed for 3–8 independent biological replicates, with two cDNA syntheses and RT-PCRs for each. Numbers shown are transcript ratios for each group indicated (in the column header) relative to transcript levels of the same genes in near-isogenic N2DRM controls. All C(t) data (threshold cycle numbers) were normalized to the mean values for three control genes (β-actin, T08G5.3, and Y71D11.3) that did not change among the strains/groups tested. Changes that were at least nominally significant (*P*<0.05) are emphasized with bold font.

The *C. elegans* genome contains at least 38 genes encoding putative insulinlike peptides (ILPs), although functional roles in the initiation of IIS have been established for rather few ILPs [12]. Genes encoding six ILPs, known or presumed ligands of the DAF-2 insulin/IGF-1 receptor, were selected for study based on prior evidence of a biological function, as cited in WormBase (www.wormbase.org). Five of these (including genes for known IIS antagonists INS-1 and INS-18) were upregulated 3- to 10-fold in *age-1(mg44)* F2 adults, whereas the *ins-7* ILP gene—an established ILP-receptor agonist for which RNA interference (RNAi) extends life [39]—had roughly half the wild-type transcript level. In addition, two positive targets known to be induced by DAF-16/FOXO [24],[40] serve to confirm its enhanced activity in *age-1(mg44)* adults: *sod-3*, encoding mitochondrial superoxide dismutase-3 (up 8×), and R11A5.4, which encodes phosphoenolpyruvate carboxykinase (PEPCK), a key activator of gluconeogenesis (up 8.5×).

For the most part, this profile of IIS transcripts is consistent with concerted silencing of the IIS pathway in *age-1(mg44)*, leaving DAF-16 in its active state, unphosphorylated at sites that would exclude it from the nucleus. Thus, in *age-1(mg44)* F2 adults, insulinlike peptide genes that antagonize IIS are upregulated while *ins-7* (the only confirmed IIS agonist) is downregulated. Genes supplying the IIS kinase cascade that inactivates DAF-16/FOXO (*daf-2*, *age-1*, *sgk-1*, and *pdk-1*) are all downregulated, whereas *ist-1*, encoding a homolog of mammalian IRS-1 and IRS-2 [41] which serve as convergence points for many inhibitory inputs from other signaling pathways [41],[42], is upregulated. Moreover, *aak-2*, the sole AMP-dependent kinase (AMPK) gene implicated in DAF-16/FOXO activation [43],[44], and shown to extend lifespan when over-expressed [45], is upregulated in *age-1(mg44)* F2 adults (Table 1). Apparent exceptions include the low transcript levels for two genes that antagonize IIS: *daf-18*, encoding the PIP_3_ 3-phosphatase PTEN, and *par-5*, encoding one of several 14-3-3 proteins that sequester IIS-phosphorylated DAF-16—genes that arguably are superfluous in the absence of PIP_3_ and IIS-mediated phosphorylation of DAF-16, respectively.

#### Other signal transduction genes

In view of the global suppression of kinase activities (Figure 1), we extended the transcriptional assay panel to include non-IIS kinases and other signal-transduction genes (Table 1). The TGF-β and ERK/MAPK (mitogen-activated protein kinase) signaling pathways, known to cross-talk extensively with IIS [46], are thoroughly silenced, with 3 and 6 genes (respectively) downregulated by more than 3-fold. In contrast, transcripts are elevated for the *daf-7* gene, encoding one of four known TGF-β peptides in *C. elegans*. The nutrient-responsive AMPK/TOR pathway is largely silenced, although its translational output, *rsks-1*/S6K, is not. The two AMPK genes, *aak-1* and *aak-2*, clearly serve distinct functions (in view of differing RNAi effects), and may diverge in their roles with respect to inhibition of the TOR complex and IIS (via IST-1/IRS-1/2), or activating phosphorylation of DAF-16/FOXO [43]–[45]. The p38/MAPK pathway, which also interacts with IIS [47], is ambiguous in that only one of the three p38/MAPK genes is significantly downregulated (*pmk-2*, down 5-fold, *P*<0.005), while the other two are much less affected and *atf-2*, mediating transcriptional output from this pathway, is induced (Table 1). A similar conflict of effects is evident in the JNK/MAPK pathway, wherein *jnk-1* (encoding Jun N-terminal kinase 1) is induced in *age-1(mg44)* F2 adults, but one of its targets, *jun-1* encoding the JUN transcription factor, is modestly downregulated. This suggests that the critical activation target of JNK-1 (in the context of *age-1* longevity) is not JUN, but DAF-16/ FOXO (see also [48]).

Altogether, this targeted gene survey indicates widespread silencing of signal-transduction genes in *age-1(mg44)* F2 adults, with significant declines relative to N2DRM (*P*<0.01) for 23 of the 47 genes tested, and increases for 12 genes. The declines imply attenuation of IIS, TGF-β, and ERK/MAPK pathways, and either silencing or redirection of the AMPK/TOR nutrient-sensing pathway. Two pathways implicated in immune and stress responses, p38/MAPK and JNK/MAPK, do not appear to have all outputs inhibited at the transcript level. The bias toward inhibition over induction cannot be a reflection of global transcriptional silencing, because all data were normalized to the mean of three control genes (β-actin, T08G5.3, and Y71D11.3) that did not change among the strains tested. However, the bias is consistent with the results of a microarray survey (Ayyadevara *et al.*, submitted) which indicated that differential gene expression in strong *age-1* mutants reflects primarily transcriptional suppression targeting a few hundred genes, largely or entirely specific to those alleles.

#### Expression changes in *age-1(mg44)* F2 adults do not simply recapitulate a younger adult state in wild-type worms

**Figure 4:**
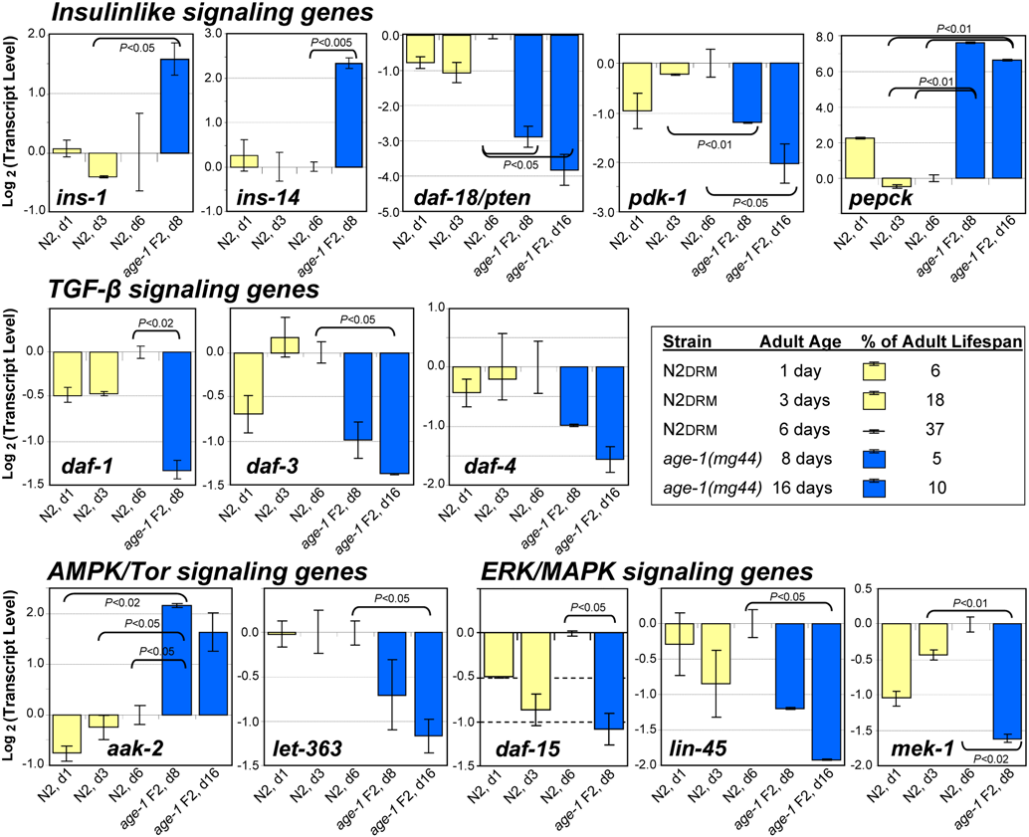
Most transcriptional changes in *age-1(mg44)* F2 adults remain even after scaling for lifespan. Transcript levels were assayed by real-time polymerase chain reaction (RT–PCR) as described in the legends to Figure 3 and Table 1. Within each histogram, means±SEM are shown on a log(2) scale for steady-state transcript level, comparing wild-type N2DRM adults at three ages to *age-1(mg44)* F2 homozygotes at one or two adult ages. All transcript values were first normalized to β-actin mRNA (to adjust for variation in RNA inputs), and then each biological group was normalized to the mean value for N2DRM day-6 adults (to correct for run-to-run variation in C(t) values). Ages are measured from the L4/adult molt; a table (inset) indicates the percent of *adult* lifespan represented by each age. To monitor longitudinal changes, two biological preparations of each group were assessed (fewer than used for Figure 3 or Table 1). Significance of differences was ascertained by two-tailed Behrens-Fisher *t*-tests, appropriate to samples of unequal or unknown variance. The transcript levels observed in this experiment differ from the means seen in previous experiments (*e.g.*, Table 1), but those differences are not significant.

Might the observed differences in gene expression reflect the considerable differences in physiological age (or in chronological age) among the young-adult worms being compared? Gene expression in Drosophila lines, varying in longevity by up to threefold, was reported to fall into two classes: some genes alter transcript levels over time, independent of lifespan, while others show expression patterns that scale with lifespan [49]. Of the genes that appeared strongly modulated in *age-1(mg44)* F2 adults, a subset was chosen to represent four signaling pathways, and assessed in wild-type N2DRM adults at several adult ages, as well as one or two ages of *age-1(mg44)* F2 adults. This time series (Figure 4) demonstrates that only a few of the expression changes observed in very long-lived *age-1* worms could be interpreted as reflections of their physiologically youthful state relative to wild-type worms. In particular, expression in *age-1(mg44)* F2 worms at 8 and 16 days of adult age (5 and 10% of their mean adult life spans) did not overlap the levels seen in N2DRM worms at 1, 3 or 6 days adult age (6, 18 or 37% of life span), for the genes *ins-1*, *ins-14*, *daf-18*, *pepck*, *daf-1*, *daf-4*, *aak-2*, or *let-363*. Thus, the differences in their expression mirror genotype rather than aging. In contrast, scaling to lifespan largely eliminates the difference between strains for two genes of the ERK/MAPK pathway (*daf-15* and *mek-1*), while intermediate outcomes were seen for *pdk-1*, *daf-3*, and *lin-45*.

#### Silencing involves both *daf-16*–dependent and –independent routes

Of 35 nominally significant (*P*<0.01) transcriptional differences between *age-1(mg44)* F2 and N2DRM adults, 25 were completely reversed (to within 20% of the N2DRM level, or over-reverted) by mutations inactivating *daf-16*; a further 9 genes showed partial reversion, while two, *daf-2* and *pmk-2*, did not revert at all. Four instances of partial or negligible reversion (*ins-14*, *daf-2*, *pmk-2* and *pmk-3*) entail expression levels for *daf-16(mu86); age-1(mg44)* adults that differed significantly from N2DRM (each *P*<0.01; combined *P*<10^−9^). Results were similar for the *m26* and *mu86* alleles of *daf-16*; Table 1 shows only data for the *mu86* allele in which most *daf-16* exons are deleted. These data demonstrate that *age-1(mg44)* F2 effects on transcription are primarily mediated by DAF-16/FOXO, but are also supplemented by DAF-16-independent mechanisms—paralleling the conclusions drawn from earlier data on *daf-2* mutants [50].

#### Expression changes differ between F2 and F1 *age-1(mg44)* homozygotes

Transcriptional modulation is also seen in first-generation (F1) *age-1(mg44)* adults, for which inductions occasionally exceeded the level seen at F2. In fact, the three highest induction factors were observed in F1 adults, for *ins-5* (24×), *ins-14* (27×), and *atf-2* (44×), with *ins-6* (11×) also showing substantially higher transcript levels in F1 worms than in F2. However, expression of 21 genes is altered 2- to 8-fold *more* in F2 worms than in F1 (*e.g.*, *ist-1*, *vps-34*, *daf-18*, *sod-3*, *smk-1*, *sir2.1*, *aak-1* and *daf-15*), while 6 genes were roughly equal at the two generations (*age-1*, *sgk-1*, *daf-3*, *let-60*, *mek-1*, and *gsk-3*). Clearly, the consequences of maternal protection differ among these genes.

#### Comparison to other IIS mutants and to dauer larvae

F2 *age-1(mg44)* adults generally show far more pronounced transcriptional effects than the weaker *age-1* allele, *hx546* (Figure 3 and Table 1). Only two of 47 tested genes (4%) changed significantly (at *P*<0.01) in *age-1(hx546)* adults: *ins-1* (up 16× over N2DRM) and *ins-7* (up 5.3× in *hx546*, but down 2-fold in *mg44*-F2 adults). This is in stark contrast to 35 significantly differential genes (74%) seen in *mg44*-F2 adults—underscoring the quite atypical properties of the stronger *age-1* allele. The widely studied *daf-2(e1370)* strain was tested for transcript levels of 17 genes, of which only 4 (24%) differed significantly from N2DRM (at *P*<0.01; see Table S2). These four genes (*ins-1*, *sgk-1*, *daf-7* and *aak-2*) were all upregulated in the *daf-2* mutant. Like *age-1(hx546)*, *daf-2(e1370)* is a temperature-sensitive mutant of much weaker effect than the *age-1* nonsense mutants; both give a life extension of about twofold in our hands, after outcrossing into the N2DRM background (data not shown). Both *daf-2(e1370)* and *age-1(hx546)* are upregulated for most signaling components tested in the IIS and TGF-β pathways, whereas nearly all of these genes (all except *ins-1*, encoding an IIS antagonist, and *daf-7*, encoding one of four TGF-β ligands) are strongly suppressed in *age-1(mg44)* F1 and especially F2 adults (Table S2).

F2 *age-1(mg44)* adults differed significantly from dauer larvae for 43% (20/46) of the signal-transduction genes tested for both; the same number (20) had transcript levels that were within twofold of those in dauer larvae (Table 1)—suggesting that these two very long-lived populations achieve resistance to aging through distinct but partially overlapping gene-expression strategies.

The transcript-level changes observed in *age-1(mg44)* F2 adults were cross-checked against those previously reported for either *daf-2(e1370)* adults, wild-type dauer larvae, or both [21], [24], [51]–[53]. Few commonalities were found between the present data and these gene lists, which comprise the most highly and significantly altered genes in microarray surveys. The differences may in part reflect the greater sensitivity of RT-PCR to detect and quantify low-abundance transcripts; however, data in Tables 1 and Table S2 imply that transcriptional shifts for *age-1(mg44)* F2 adults differ both qualitatively and quantitatively from those seen in other mutants.

### Functional consequences of transcriptional suppression in *age-1(mg44)* worms

**Figure 5:**
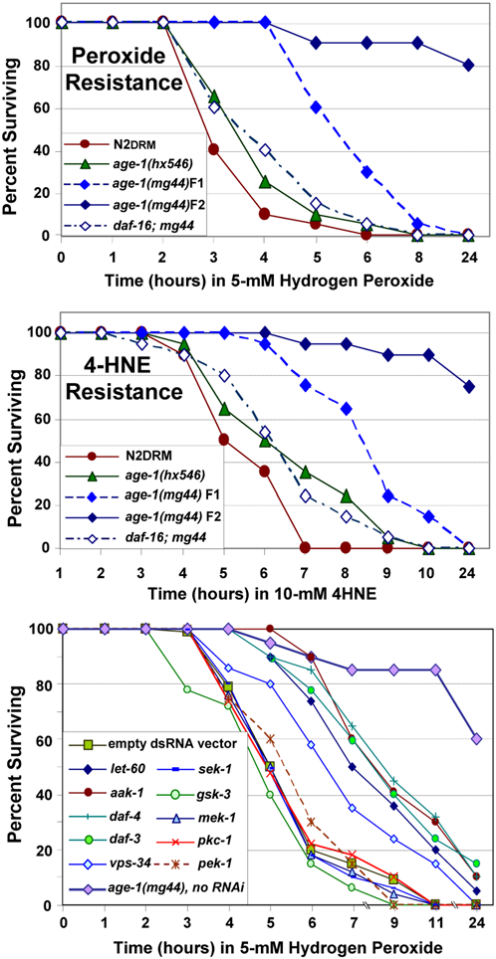
Survival of oxidative and electrophilic stresses. N2DRM worms were exposed to (A) 5-mM hydrogen peroxide (H_2_O_2_), or (B) 10-mM 4-hydroxynonenal (4-HNE). Stress survivals began on adult day 3–4 and worms were maintained in liquid medium, 20±0.3°C, without bacteria. Results shown were replicated in independent experiments. Statistics (log-rank test, each *n* = 40–50, both A and B): *age-1(mg44)* at F2 *vs.* F1 generation: *P*<10^−7^; *age-1(mg44)* F1 *vs.* any other strain: *P*<10^−3^; *age-1(mg44)* F2 *vs.* any other strain or group: *P*<10^−9^; *age-1(hx546) vs.* N2DRM: *P*<0.004; *daf-16(mu86); age-1(mg44) vs.* N2DRM: *P*<0.005. (C) Functional consequences of suppressing signaling genes downregulated in *age-1(mg44)* F2 adults. N2DRM worms were picked on day 1 of adulthood, and maintained on dsRNA-expressing *E. coli*, at 20°C, for 3 days. RNAi extension of 50^th^-percentile H_2_O_2_ survival, as percent gain over N2DRM fed on bacteria carrying empty expression vector (significance, by Gehans log-rank test), is as follows: *vps-34*, 26% (*P*<0.03); *let-60/RAS*, 40% (*P*<0.001); *daf-3*, 49% (*P*<0.0003); *aak-1*, 49% (*P*<0.00003); *daf-4*, 57% (*P*<0.0001). All significant effects except *vps-34* were confirmed (each *P*<0.001) in independent experiments. Peroxide survival of F2 *age-1(mg44)* day-62 adults, without RNAi, exceeded the N2DRM controls by 5.6-fold (*P*<10^−6^), at the 60^th^ percentile (the lowest survival observed for the F2 group).

F2 *age-1(mg44)* adults, which are 4- to 5-fold longer-lived than F1 adults (comparing data of [8],[29] to [30]), also outperform their parents with respect to resistance to oxidative and electrophilic stresses (Figure 5A and 5B). Relative to N2DRM controls, survival in 5% hydrogen peroxide is extended 2-fold in F1 adults, but 10-fold in their F2 progeny. Because *age-1(mg44)*-F2 adults barely reached 20% mortality after 24 h, by which time 100% of worms had died in all other groups, survival time is here compared at a threshold of 20% mortality. Survival of an electrophilic stress, 4-HNE (similarly defined as time to 20% mortality) increased 1.6-fold in *age-1(mg44)* F1 worms but 5-fold at F2, with reference to N2DRM. Although resistance to these stresses is restored almost to wild-type levels in double mutants with *daf-16*, we note that reversion is not quite complete, whether using the weaker *daf-16(m26)* allele [30] or the *daf-16(mu86)* deletion allele (Figure 5A), indicating that such stress-resistance traits are mediated in part by a DAF-16-independent pathway. These results parallel the incomplete reversion, in *daf-16; age-1(mg44)* double mutants, seen for *in vitro* kinase activity and phosphoprotein levels (Figure 1) and for transcript levels of several genes (Table 1).

Most or all of the *age-1(mg44)*-downregulated genes are essential for nematode growth and development. That is, double-stranded RNAs (dsRNAs) targeting them, administered to developing *C. elegans*, produce embryonic lethality or larval arrest [54]–[56]. The impact of such knockdown, however, has thus far remained largely untested in adults. Because resistance to oxidative stresses is a common feature of many long-lived *C. elegans* mutants [57], and in particular parallels longevity in the *age-1* allele set studied here (Figure 5 and [30]), we employed it as a short-term assay to evaluate the contribution of individual-gene downregulation, to the exceptional survival of *age-1(mg44)*-F2 adults in both benign and toxic environments.

Hydrogen peroxide resistance was measured in duplicate experiments, for wild-type N2DRM worms that had been exposed to dsRNA-expressing bacteria targeting 10 genes for which transcript levels are markedly reduced in *age-1(mg44)*-F2 adults. Genes were selected from among those not directly involved in the IIS pathway, but representing a variety of other signaling pathways, and for which RNAi constructs were available from the Ahringer library [56]. *E. coli*, either harboring an empty-vector control or expressing one of 10 gene-targeted dsRNA species, were fed to mature adults (days 3 through 6 after the L4/adult molt) so as to preclude effects on development. Survival curves, during subsequent exposure to 5-mM H_2_O_2_, are shown in Figure 5C. RNAi for four of the ten genes (encoding a transcription factor and three components of distinct protein-kinase signaling cascades) produced highly significant gains in peroxide survival (each *P*<0.001), and a fifth dsRNA exposure offered marginally significant protection (*vps-34*, *P*<0.03). The remaining five dsRNA treatments had no discernible effect on survival, compared to worms exposed only to the empty expression vector. The above results were reproduced in an independent experiment, with the same four genes attaining *P*<0.001, while *vps-34* achieved *P*<0.08.

Genes (and encoded proteins) for which RNAi knock-down conferred a protective effect were *daf-3* (SMAD transcription factor) and *daf-4* (TGF-β receptor, a Ser/Thr kinase), both involved in TGF-β signaling; *aak-1* (AMP-dependent protein kinase 1), part of the AMPK/TOR pathway; *let-60* (RAS-family GTPase activating MAPK), part of the ERK-MAPK pathway, and *vps-34* (class-III PI3K_CS_), involved in vesicular trafficking and autophagy. None of these individual RNAi effects matched the peroxide survival of untreated *age-1(mg44)* F2 adults at 62 days of adult age (large diamond symbols, Figure 5C). These data demonstrate that transcript-level changes seen in *age-1(mg44)* F2 homozygotes favor oxidative-stress survival. They may also contribute incrementally to the greatly enhanced longevity of F2 homozygotes, but we have not been able to confirm such effects. When begun at the end of larval development, RNAi to *aak-1* and *daf-4* extended survival by 7–11%, while *let-60* dsRNA reduced it by ∼12% (data not shown). Such small effects on life span require large groups to reach significance; moreover, significance in one experiment provides little assurance that independent replicates will attain significance. This may reflect the low statistical power inherent to survivals of modest size, and/or inability to control environmental variance among experiments.

## Discussion

First-generation homozygotes for the *age-1(mg44)* mutation develop normally into fertile adults at 15–25°C, and display stress resistance and life extension typical of many IIS mutants [8],[14],[29]. In contrast, their progeny—second generation homozygotes—develop slowly at 15–20°C, to form infertile adults that are far more stress resistant and at least four-fold longer lived than other IIS mutants [30]. Maternal protection (oocyte carryover of *age-1* mRNA, AGE-1 kinase or its PIP_3_ product, synthesized by the heterozygous parent) is thought to blunt both the stress-resistance and longevity traits to approximately those of a weaker *age-1* or *daf-2* allele [30]. We here show that total kinase activity is also attenuated more severely in *age-1(mg44)* adults at the F2 than the F1 generation (see Figure 1C and Figure S2), although several kinase substrates remain unaffected by this mutation (Table S1 and Figure S2). At the expression level, the situation is more complex: the majority of tested genes are most strongly affected in the F2 generation of *age-1(mg44)*, whereas others show transcript effects in F1 adults that equal, exceed, or even oppose those observed in F2 adults (Table 1).

### Consequences of altered gene expression for IIS and other pathways

Transcriptional effects within the IIS pathway seem fully consistent with impaired insulinlike signaling, which might be expected to further augment survival through the same mechanisms employed by weaker IIS mutations. Moreover, repression of class-II and class-III PI3K catalytic-subunit genes (Table 1) would impede formation of PI(3)P and PI(3,4)P_2_, suppressing alternative routes to PI(3,4,5)P_3_. Increased expression of *aak-2* contributes to activation of DAF-16/FOXO, further opposing IIS (which inhibits this transcription factor) and increasing life span [43],[44].

In addition to effects on IIS genes, however, *age-1(mg44)*-F2 adults also show striking transcriptional attenuation of several other signal transduction pathways that interact with IIS and with one another. TGF-β endocrine/paracrine signaling is active in development, and modulates several other signaling pathways including p38/MAPK and ERK/MAPK [46]. Both *daf-1* and *daf-4*, encoding type-I and -II TGF-β receptors, respectively, are downregulated 4- to 5-fold in *age-1(mg44)*-F2 adults. Expression is also reduced 3-fold for *daf-3*, encoding a co-SMAD transcription factor deployed by several pathways including TGF-β. Perhaps in partial compensation for this signaling downregulation, the *daf-7* gene encoding a TGF-β-family ligand/agonist is 5-fold upregulated. Silencing of TGF-β signaling, by RNAi directed at *daf-3* or *daf-4*, improves survival in the presence of hydrogen peroxide, consistent with a prior observation that *daf-1*, *-4* and *-7* mutants are long-lived [46].

AMPK/TOR signaling has been implicated in innate immunity and stress responses. Although it remains controversial whether the primary response is to the microbe or to the stress it causes [47], both could be secondary to its role in nutrient sensing [37],[43]. AAK-1 and AAK-2 are regulated by the PAR-4 transcription factor [37],[58],[59], and *aak-1* knockdown by RNAi confers resistance to oxidative stress (Figure 5C). Inhibition of the *C. elegans* TOR pathway confers stress resistance and extends life span [60],[61].

ERK/MAPK signal-transduction is essential for many developmental processes; because the constituent genes are also expressed in adult nematode tissues, they are presumed to have post-developmental functions not yet defined [62]–[64]. All six genes tested in this pathway are markedly downregulated, by 3- to >6-fold (Table 1), and RNAi inhibition of *let-60* (encoding a RAS membrane co-receptor that initiates ERK/MAPK signaling) significantly improves survival of oxidative stress (Figure 5C).

### Rationale, anomalies, and resolutions

The most dramatic effects of gene mutations on life span have involved hypomorphic (loss-of-function) mutations, and the genes affected have been termed aging “master genes”. The genes encoding IIS components provide the best-studied example. IIS, in common with many “master genes” and essentially all signaling pathways, regulates numerous other genes. In the case of IIS, a number of these are modulated in ways that are protective, or otherwise conducive to long life, such as upregulation of GSTs and other detoxification genes, which are among the “foot soldiers” of longevity assurance [65],[66]. However, we should not expect all such downstream consequences to confer uniformly pro-longevity effects; each gene is likely to serve several “masters”, and its level of expression will depend on the genetic, environmental, and signaling context. In keeping with this perspective, the downstream manifestations of longevity assurance genes are far less conserved, both in evolution and between distinct physiological states of a given species, than are the over-arching pathways and the functions they serve [67].

Improved stress resistance and survival of *age-1(mg44)* F2 worms, apparently arising from transcriptional attenuation of signaling pathways presumed to be protective, poses an intriguing paradox. These pathways, activated by nutrient deficiency, pathogens, or growth factors, have been reported to cross-talk with IIS at diverse levels [19], [43]–[47],[68],[69]. This suggests a complex fabric of signaling interactions, for which the impact of silencing multiple components cannot be predicted. Moreover, signaling that promotes survival in a variable or hostile setting may entail energy costs and harmful side-effects that would be unwarranted in a constant, pathogen-free environment with abundant food. An organism that avoids the deleterious aspects of these surveillance systems may thus reap survival benefits under benign conditions.

In several instances, the expression changes seen in strong-*age-1* mutants appear to oppose their longevity or stress-resistance, based on the effects of down- or upregulation previously reported for the same genes. For example, *age-1(mg44)* F2 adults downregulate ***daf-18***, which encodes the PIP_3_ 3-phosphatase, PTEN. This would be expected to elevate the steady-state level of PIP_3_, thereby enhancing IIS and *reducing* longevity of normal worms. However, in the absence of AGE-1/PI3K_CS_ kinase activity, there may be little or no PIP_3_ substrate on which PTEN could act. A second example is downregulation in *age-1(mg44)* F2 adults of ***skn-1***, encoding a transcription factor responsive to oxidative damage and regulated via IIS [70]–[72]. Reduced expression of *skn-1* seems at odds with increased oxidative-stress resistance and longevity; however, these very long-lived worms may generate lower levels of reactive oxygen species, thereby reducing *skn-1* induction. RNAi to ***vps-34*** (encoding a class-III PI3K_CS_ required for vesicular trafficking and autophagy [73]) was recently shown to block life extension of *eat-2(ad1116)* and *daf-2(mu150)* mutants, although not of wild-type worms [58]. Autophagy is induced by TOR deficiency [58], and several TOR signaling components are downregulated in F2 worms (Table 1). Considering this, autophagy should be at least moderately induced in those worms, and its absence would not account for low expression of *vps-34*. These results argue against any direct role of *vps-34* attenuation in the exceptional longevity of *age-1(mg44)* F2 worms. The possibility remains, however, that *vps-34* downregulation could reinforce PIP_3_-depleting effects of a strong *age-1* mutation. Downregulation in *age-1(mg44)* worms, of transcripts for ***let-60*** **/RAS** and five other members of the ERK-MAPK cascade, might be expected to oppose additional life extension beyond that typical of IIS mutants, because a *let-60* gain-of-function mutation enhances *daf-2* life extension [74]. RNAi targeting ***smk-1***, encoding a transcriptional coactivator shared by DAF-16 and PHA-4 [75], reduces stress-resistance and lifespan of *daf-2(e1370)* worms [76], whereas the effect on wild-type worms is controversial [34],[76]. Although *smk-1* knockdown impairs *sod-3* expression in *daf-2* worms [34], we found 9-fold elevation of *sod-3* transcripts in the face of a 72% drop in *smk-1* expression in *age-1(mg44)* (Table 1). Finally, ***sir-2.1*** overexpression was reported to extend lifespan, and knock-down to shorten it [77],[78], whereas we found almost 4-fold downregulation of *sir-2.1* in *age-1(mg44)*-F2 adults.

Although contradictory in the context of extreme stress resistance and longevity, all six of these “exceptions” are also mirrored, in most cases to a lesser degree, in dauer larvae (Table 1), a robust state of developmental arrest that can endure for months without reducing adult life span [1],[79],[80]. This raises the possibility that for these genes, the effects of downregulation are context-dependent, and may be beneficial in worms that are already highly protected from stress and aging. Alternatively, these expression changes may follow from regulatory mechanisms shared by *age-1(mg44)* adults and N2 dauers, and yet work in opposition to their robustness. This is plausible in the case of a severe loss-of-function mutant, effects of which are not orchestrated, but is difficult to reconcile with a highly-evolved alternative developmental state such as the dauer larva. Perhaps, rather than a single coherent program, the patterns we observe reflect aberrant triggering in the adult of one or more regulatory mechanisms that are normally utilized in developmental or metabolic regulation. This particular combination of mechanisms could be the serendipitous result of a profound alteration in PIP_3_ levels which in turn impacts multiple pathways.

### PI3Kcs-mutant effects are largely, but not entirely, mediated by DAF-16/FOXO

The expression profile of *age-1(mg44)* worms depends largely on DAF-16/FOXO, consistent with prior evidence that *C. elegans* IIS operates mainly through this transcription factor, impacting several hundred target genes [2],[21],[22],[29],[52],[81]. Although DAF-16/FOXO has been regarded largely as a transcriptional activator [21],[82], it also effects negative regulation of many genes [24]. In our selected panel of genes, two-thirds (22/33) of the DAF-16-mediated effects of *age-1(mg44)* mutation involve reduced transcript levels, indicating that silencing prevails for gene transcripts that encode kinases and other mediators of intracellular signaling. Twenty-eight genes (of the 33 for which transcripts appear to be primarily regulated via DAF-16/FOXO) were mapped for DAF-16 binding sites within 5 kb upstream of the initiation codon (Table 1). Of these, 21 (75%) have exact matches to one or both of the two known consensus sites, GTAAA(^C^/_A_)AA and CTTATCA. Genes lacking such sites may be indirect targets of DAF-16/FOXO, but considering that those motifs occur at almost the same frequency in the genome at large [81], as in DNA immunoprecipitated with antibody to DAF-16/FOXO [52], it is possible that precise motif matches are neither necessary nor sufficient for DAF-16 binding. In other words, near-match sequences might be able to bind DAF-16, while even perfect-match motifs may require additional features in nearby DNA.

### Implication of a hybrid feedback loop with kinase and transcriptional components

**Figure 6:**
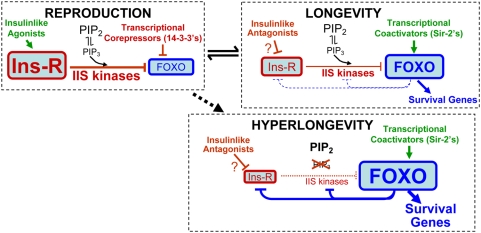
Model of the IIS “molecular switch.” Three proposed system states are depicted, reflecting the balance between kinase signaling and transcriptional feedback suppression: *reproductive mode*, in which kinase signaling predominates, activated via the insulin/IGF-1 receptor (DAF-2, here labeled Ins-R); *longevity mode*, wherein FOXO (DAF-16) prevails and suppresses kinase transcription; and *hyperlongevity mode*—in which switching between the first two modes is blocked, thus strongly favoring survival but with no possibility of resuming reproduction. State transitions are normally triggered by signaling modulators (e.g., insulin-like peptides [12] or SIR-2/14-3-3 complex [77]), to which *age-1(mg44)*-F2 mutants (“hyperlongevity” state) cannot respond.

It is surprising that hyperactivation of DAF-16/FOXO in *age-1(mg44)* F2 adults silences essentially the entire IIS pathway. This implies a positive feedback loop, in which DAF-16/FOXO imposes transcriptional silencing on the very kinases that would inhibit its own nuclear localization and hence access to target genes (Figure 6). We propose that second-generation *age-1(mg44)* homozygotes are trapped in a nonadaptive state, incapable of responding to diverse environmental and internal signals. This apparent paradox, that *failure* of adaptive mechanisms greatly extends lifespan, is easily explained because those mechanisms maximize Darwinian fitness – transmission of genetic alleles to ensuing generations – rather than individual survival [83],[84].

When IIS kinase signaling predominates (the *reproductive state*), it suppresses DAF-16/FOXO activity. Activation of PI3K favors PIP_3_ production and AKT activation, both of which promote cell proliferation [18],[31],[85]. However, IIS can switch to a second, functionally distinct, state: when kinase signaling is weak, DAF-16/FOXO becomes activated. As we have demonstrated, active DAF-16/FOXO transcriptionally silences its own upstream regulatory kinases, which otherwise would have impeded DAF-16/FOXO action by preventing its nuclear localization. Therefore, the low-signaling, *longevity state* of IIS is self-sustaining. Biologically, this state promotes dauer formation during development, or life-extension and delayed reproduction in the adult (reviewed in [4]). Signals that inhibit IIS kinases or augment DAF-16/FOXO action, if sufficient, trigger a switch from reproductive to longevity state in which DAF-16/FOXO promotes somatic protective mechanisms (Figure 6). However, exiting the stable longevity mode requires a shift in the balance of inputs that govern the positive feedback loop. Such inputs may include insulin-like peptide agonists and antagonists, hormones, pheromones, transcriptional co-activators and co-repressors of DAF-16/FOXO such as SIR-2 and 14-3-3 proteins, and nutrient- and stress-sensors signaled though other kinase pathways (*e.g.*, MAPK, JNK and AMPK) that cross-talk with IIS. Combined, these two normal states of the IIS pathway (reproductive and longevity) constitute a “flip-flop” circuit with opposing kinase-cascade and transcriptional signals (Figure 6).

The concept of a “genetic switch” for dauer formation is not new [1],[86],[87], and has even been demonstrated to constitute a bistable feedback loop [21],[87]. Nevertheless, a dual-level (kinase/transcriptional) feedback mechanism had not previously been proposed or described. Any such “flip-flop” circuitry allows the organism a simple binary choice in response to its environment: early reproduction under benign conditions, or postponed reproduction and extended survival in harsher conditions. Mutational disruption of IIS forces dauer formation, irrespective of environment, although larvae with temperature-sensitive mutations can mature at lower temperatures into long-lived adults. Recovery from the dauer state requires that pro-reproductive-state kinases retain partial function, so that favorable signals (restoration of food, absence of stress and crowding) can reset the switch to the reproductive mode; this requirement is demonstrated by the impaired post-dauer recovery of IIS-defective mutants [86]. Nonsense mutations truncating AGE-1 produce an extreme phenotype that forfeits this option, while acquiring a distinctive transcriptional profile and greatly enhanced survival. Details of the mechanism or mechanisms, by which elimination of PI3K activity blocks exit from the longevity mode and promotes extreme longevity, remain to be elaborated. Features described in this report, which may contribute, include transcriptional silencing of upstream and collateral signaling components, and accompanying loss of multiple kinase activities. Infertile mutants may thus reveal new strategies to extend life well beyond the limits imposed by natural selection, which of course requires reproduction. In view of the striking evolutionary conservation of the IIS pathway, and the emerging parallels between inter-pathway cross-talk in nematodes and mammals [16],[19],[47],[68],[69], the mechanistic insights afforded by very long-lived worms are likely to also apply to insulin and IGF-1 pathways of mammals.

## Materials and Methods

### Strains

Nematode strains, supplied by the Caenorhabditis Genetics Center (CGC, Minneapolis), or derived in our laboratory from CGC strains, were maintained at 20°C on 0.6% peptone NGM-agar plates seeded with *E. coli* strain OP50, as described [88]–[90].

### Stress resistance

Assays of survival in the presence of 5-mM hydrogen peroxide or 10-mM 4-hydroxynonenal were modified from Ayyadevara *et al.* [91]. Wild-type (N2DRM) worms were assayed at day 3–4 of adulthood (∼6 d post-hatch). For RNAi experiments, day-1 adults were washed in S-buffer [92] and transferred to nutrient-agar plates seeded with dsRNA-expressing *E. coli* [56]. After 3 days at 20°C, 20 worms from each RNAi treatment were transferred to 24-well plates containing 300 µl of S Buffer plus 5 µg/ml cholesterol, supplemented, as indicated, either with 5-mM H_2_O_2_ (freshly diluted from 30% H_2_O_2_, Sigma) or with 10-mM 4-HNE (freshly obtained by acid hydrolysis of 4-HNE dimethylacetal which was synthesized according to [93]. Survival was scored as described [30],[89].

### *In vitro* phosphorylation assay

Worms grown at 20°C were quickly frozen in liquid nitrogen to preserve endogenous kinase activity. Worms suspended in 50-mM Tris pH 7.5, 80-mM β-mercaptoethanol, 2-mM EDTA, 1-mM PMSF, and Protease Inhibitor Cocktail I (CalBiochem), were ground at −78°C and sonicated (VIRTIS Virsonic 475, setting 2.5, 0°C) in six 10-s bursts interspersed with 2-min cooling periods. Kinase activity toward endogenous substrates was assessed in cleared supernatants after centrifugation (10 min, 11,000 g), representing 20 µg protein in 100 µl of buffer containing 50-mM Tris pH 7.5, 12.5-mM MgCl_2_ and (for endogenous substrates) 8–10 µCi γ-^32^P-ATP (NEN). After 1 min at 30°C, quenched samples were electrophoresed on 10% SDS-polyacrylamide gels (Invitrogen), which were stained with SYPRO Ruby (Invitrogen), and dried under vacuum. ^32^P β-emissions of bands migrating slower than a 25-kDa protein marker (Invitrogen), were imaged and quantified per lane after 6-h phosphor-screen exposure (Storm, Molecular Dynamics). Peptide arrays were incubated as above, 60 min at 30°C, but with addition of phosphatase inhibitors and 1-mM cold ATP rather than ^32^P-ATP. Arrays were then stained with Pro-Q Diamond (Invitrogen), and phosphorylation was quantified by fluorescence imaging (excitation/emission at 550/580 nm) with a ScanArray 5000 (GSI Lumonics).

### *In vivo* phosphoprotein detection and quantitation

Total protein (20 µg), extracted from each strain as above, was loaded onto NuPAGE 4–12% gradient gels (Invitrogen) and electrophoresed 1 hour at 200 V. Phosphoproteins were quantified by Pro-Q Diamond (Invitrogen) fluorescence, which depends linearly on protein concentration (>1000-fold range). Protein load was assessed by Coomassie Blue (BioRad) staining. Phosphorylated (23.6, 45.0 kDa) and unphosphorylated (14.4, 18.0, 62.6, 116.2 kDa) protein standards (BioRad) furnished positive and negative controls.

### Transcript quantitation by RT–PCR

Expression of selected genes was assessed by real-time polymerase chain reaction after an initial round of reverse transcription. Total RNA was purified from each strain (RNeasy, Qiagen), and cDNAs reverse-transcribed (SuperScript III, Invitrogen), followed by RT-PCR (Opticon2, MJ Research, using SYBR Green, Roche).

## Supporting Information

Figure S1 *In vitro* kinase activity for endogenous substrates is reduced in *age-1(mg44)* homozygotes, but not in dauer larvae. Kinase activity was assessed for N2DRM day-6 adults and eggs they produce, eggs laid by *age-1(mg44)* F1 adults, F1 adults at days 2.5 and 6.5 of adulthood, *age-1(mg44)* F2 day-1 adults, *daf-16(mu86); age-1(mg44)* adults, and N2DRM dauer larvae. Kinase activity of sonicated lysates was assessed as described in the legend to Figure 1. Only a single biological sample was assessed for each group (hence no error bars are shown); technical replicates agreed within ±20%, and replicate experiments were consistent with results shown.
(1.42 MB TIF)

Figure S2 Phosphorylation *in vitro* of peptide arrays. Fluorescence images are displayed of peptide arrays (JPT Peptide Technologies GmbH, Berlin) shown printed in duplicate blocks, stained with Pro-Q Diamond (Invitrogen) after 1-h incubation with worm homogenates containing phosphatase and protease inhibitors and 1-mM ATP. Equal protein concentrations from each strain (N2DRM, *age-1(mg44)*, and *daf-16(mu86); age-1(mg44)*) were incubated on slides, 60 min at 30°C. Note that “reversion” by *daf-16(mu86)* enhances some kinases not evident even in the wild-type (N2DRM) sample.
(0.77 MB TIF)

Figure S3 Adult *age-1(mg44)* F2 homozygotes are deficient in PI3Kcs protein. Western blots are shown, after denaturing (A) or native (B) polyacrylamide gel electrophoresis. Electrochemiluminescence (GE-Amersham “ECL-plus”) detected secondary antibody (goat anti-rabbit IgG coupled to horseradish peroxidase) to rabbit monoclonal antibodies binding the C-terminal region of *C. elegans* phosphatidylinositol 3-kinase p110 catalytic subunit, or to cytoplasmic β-actin as a loading control (Santa Cruz Biotech., Santa Cruz CA). Each lane contains 10 µg of total protein.
(0.25 MB TIF)

Table S1 Peptide phosphorylation by *C. elegans* kinases *in vitro*. Mean±SD is given for ProQ Diamond fluorescence (in arbitrary units) of synthetic peptides spotted in 2–4 arrays, after subtraction of mean background measured on negative-control spots. The kinases that phosphorylate these peptides are not known; those listed in column 2 may phosphorylate the residues underlined, based on consensus sites for the corresponding mammalian kinase.
(0.08 MB DOC)

Table S2 Gene expression data, quantified by real-time polymerase chain reaction, comparing three *age-1* mutant groups to *daf-2(e1370)* mutants. Worms in the *age-1(mg44)* F2 group are sterile adults; others are post-gravid adults. Significance of difference from N2DRM (t-test): °, nominally significant at *P*<0.05; *, *P*<0.01; **, *P*<0.001; ***, *P*<1E−4; ****, *P*<1E−5, *****, *P*<1E−6.
(0.05 MB DOC)
