# Supplementary figures and images for: Positive Feedback between Transcriptional and Kinase Suppression in Nematodes with Extraordinary Longevity and Stress Resistance
Source: PLoS Genet. 2009 Apr 10;5(4):e1000452. doi: 10.1371/journal.pgen.1000452 (PMC2661368; doi:10.1371/journal.pgen.1000452)

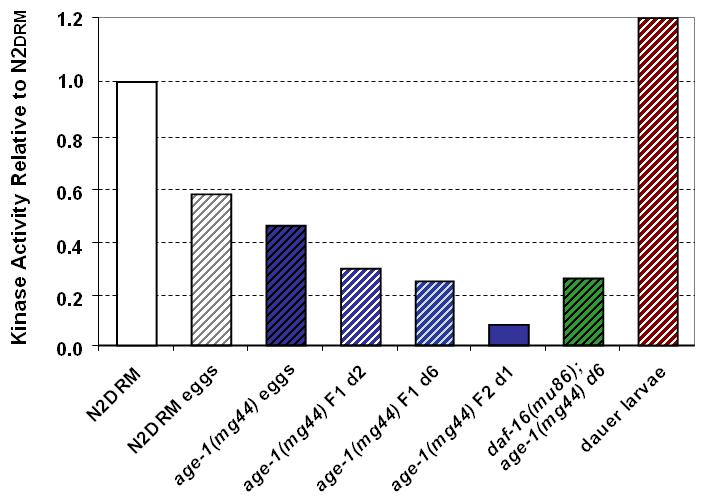

Supplement: Figure S1 — In vitro kinase activity for endogenous substrates is reduced in age-1(mg44) homozygotes, but not in dauer larvae. Kinase activity was assessed for N2DRM day-6 adults and eggs they produce, eggs laid by age-1(mg44) F1 adults, F1 adults at days 2.5 and 6.5 of adulthood, age-1(mg44) F2 day-1 adults, daf-16(mu86); age-1(mg44) adults, and N2DRM dauer larvae. Kinase activity of sonicated lysates was assessed as described in the legend to Figure 1. Only a single biological sample was assessed for each group (hence no error bars are shown); technical replicates agreed within ±20%, and replicate experiments were consistent with results shown. (1.42 MB TIF) [file pgen.1000452.s001.tif]

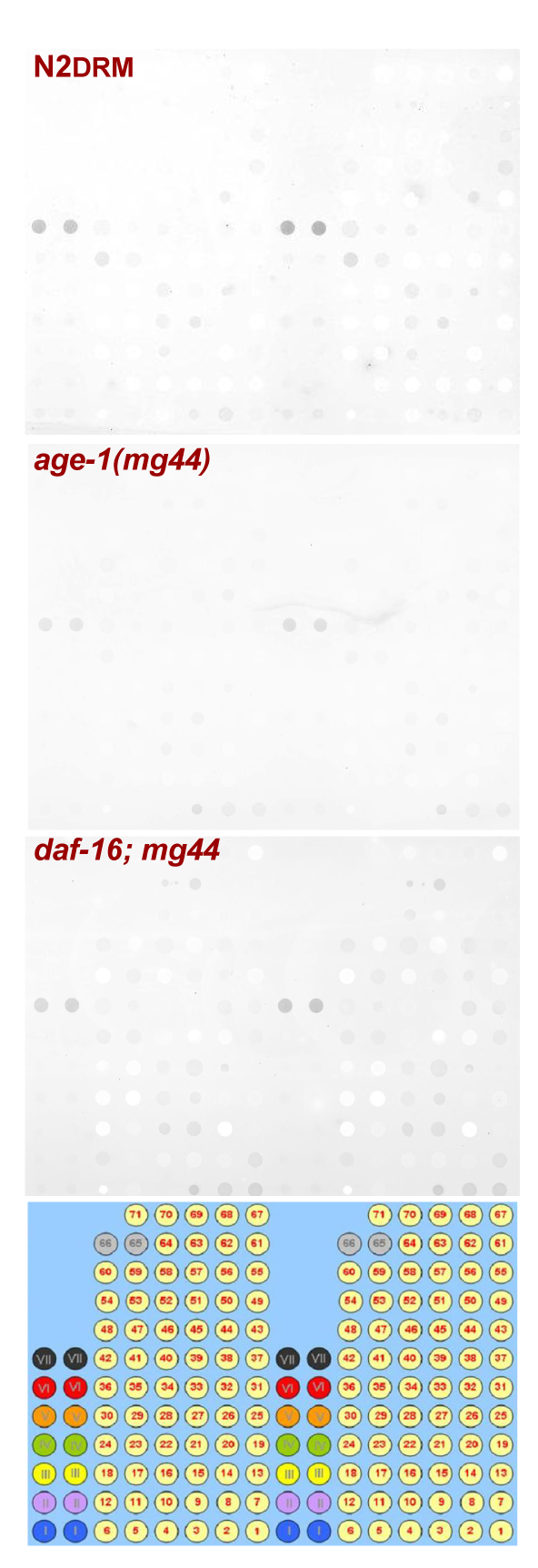

Supplement: Figure S2 — Phosphorylation in vitro of peptide arrays. Fluorescence images are displayed of peptide arrays (JPT Peptide Technologies GmbH, Berlin) shown printed in duplicate blocks, stained with Pro-Q Diamond (Invitrogen) after 1-h incubation with worm homogenates containing phosphatase and protease inhibitors and 1-mM ATP. Equal protein concentrations from each strain (N2DRM, age-1(mg44), and daf-16(mu86); age-1(mg44)) were incubated on slides, 60 min at 30°C. Note that “reversion” by daf-16(mu86) enhances some kinases not evident even in the wild-type (N2DRM) sample. (0.77 MB TIF) [file pgen.1000452.s002.tif]

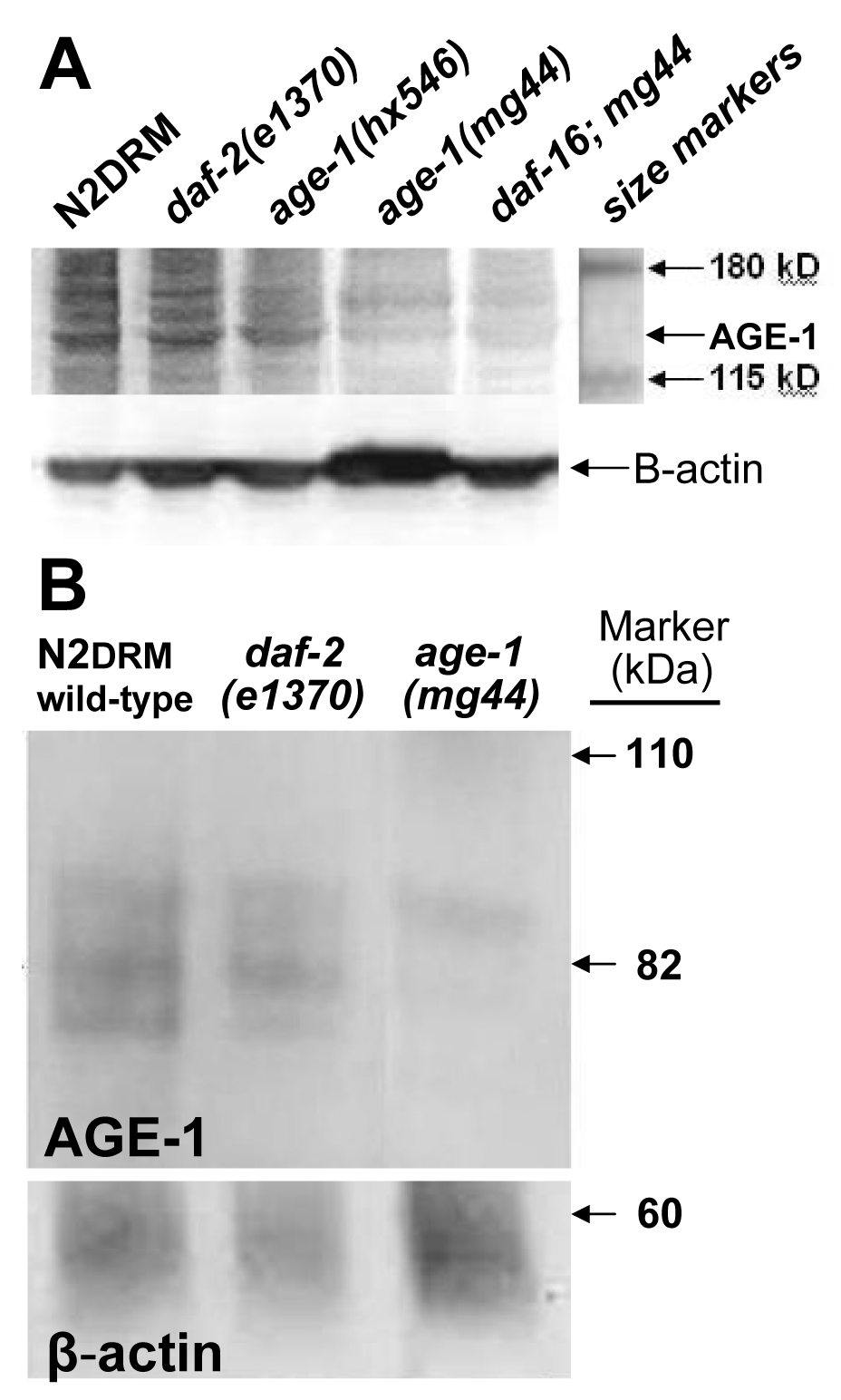

Supplement: Figure S3 — Adult age-1(mg44) F2 homozygotes are deficient in PI3Kcs protein. Western blots are shown, after denaturing (A) or native (B) polyacrylamide gel electrophoresis. Electrochemiluminescence (GE-Amersham “ECL-plus”) detected secondary antibody (goat anti-rabbit IgG coupled to horseradish peroxidase) to rabbit monoclonal antibodies binding the C-terminal region of C. elegans phosphatidylinositol 3-kinase p110 catalytic subunit, or to cytoplasmic β-actin as a loading control (Santa Cruz Biotech., Santa Cruz CA). Each lane contains 10 µg of total protein. (0.25 MB TIF) [file pgen.1000452.s003.tif]
