# Supplementary material for: Positive Feedback between Transcriptional and Kinase Suppression in Nematodes with Extraordinary Longevity and Stress Resistance
Source: PLoS Genet. 2009 Apr 10;5(4):e1000452. doi: 10.1371/journal.pgen.1000452 (PMC2661368; doi:10.1371/journal.pgen.1000452)
Supplement: Table S1 — Peptide phosphorylation by C. elegans kinases in vitro. Mean±SD is given for ProQ Diamond fluorescence (in arbitrary units) of synthetic peptides spotted in 2–4 arrays, after subtraction of mean background measured on negative-control spots. The kinases that phosphorylate these peptides are not known; those listed in column 2 may phosphorylate the residues underlined, based on consensus sites for the corresponding mammalian kinase. (0.08 MB DOC) [file pgen.1000452.s004.doc]

| # | Target Peptide / Predicted Kinase | peptide | N2DRM | *age-1 (mg44)* | *daf-16; mg44* |
| --- | --- | --- | --- | --- | --- |
| 1 | DAF-16/INSR (DAF-2) | NPWGEES**Y**SDIIAKA | 9.9±1.2 | 2.1±0.2 | 9.4±0.4 |
| 2 | DAF-16/PKC, ARK-1 | QTISHDL**Y**DDDSMQG | 11.3±1.1 | 4.2±0.3 | 8.3±0.5 |
| 3 | DAF-16/INSR, EGFR | PAIGSDI**Y**DDLEFPS | 11.6±0.9 | 3.4±0.2 | 10.6±0.5 |
| 7 | DAF-16/PKC | EPIAPPPS**Y**HELNSV | 5.4±0.6 | 0 | 7.0±0.2 |
| 15 | AKT-1 / PDK-1 | CSIDFRA**S**MISIADT | 4.8±0.6 | 0 | 6.0±0.2 |
| 16 | AKT-2 / PKC, SGK-1 | TVIERTF**Y**ADSADFRQM | 7.7±1.6 | 2.9±0.2 | 6.6±0.1 |
| 19 | IST-1 /AMPK | KIINNDT**Y**TLMGPAD | 0.4±0.6 | 0 | 2.0±0.3 |
| 20 | IST-1 /AMPK | FSRLGKN**S**ASDTKRF | 2.0±0.4 | 0 | 2.5±0.4 |
| 21 | PMK-1 /MAPKK | NWMHYTQ**T**VDVWSVG | 6.4±1.0 | 3.3±0.5 | 4.5±0.2 |
| 22 | SEK-1/EGFR (*let-23*) | DYNERPK**Y**PELLAMPF | 6.1±0.0 | 2.5±0.5 | 3.3±0.0 |
| 26 | PEK-1/EGFR (*let-23*) | LFTGEQK**Y**ECSMESC | 5.0±0.2 | 3.1±0.8 | 5.0±0.4 |
| 27 | PEK-1/ATM-1 | PLLRGTL**S**TSDPIDI | 3.1±0.9 | 0.7±0.3 | 3.4±0.4 |
| 28 | GSK-3/INSR (DAF-2) | GEKKDEL**Y**LNLILEY | 3.7±0.5 | 2.1±0.3 | 4.0±0.7 |
| 31 | AKA-1/KIN-1 (CKII) | SQESEPE**S**EPEPEPE | 1.1±0.2 | 0 | 3.6±0.2 |
| 35 | SGK-1/KIN-1, P38MK | RRFFHLE**S**PDDDENN | 2.8±0.3 | 1.0±0.6 | 4.3±1.0 |
| 36 | SGK-1/KIN-19 (CKI) | LQREKHF**S**ESRSRFY | 2.9±0.4 | 3.5±0.8 | 3.6±0.7 |
| 37 | SGK-1/unk. kinase | EYERFF**TT**EKDMIPA | 1.9±0.1 | 0 | 4.0±0.9 |
| 38 | MEK-1/INSR | QAEENSG**Y**LTLQGNR | 2.7±0.5 | 0 | 4.3±1.8 |
| 41 | DAF-3 /unk. kinase | EIPP**Y**LDP | 3.0±0.6 | 0.4±0.4 | 3.4±1.4 |
| 42 | DAF-4 /PKA,DNAPK | TEFHERL**S**LYELLKN | 2.4±0.5 | 1.0±0.6 | 2.7±0.6 |
| 44 | DAF-1/PKA | AIFLTRK**S**WEAKFDW | 3.1±0.9 | 2.7±0.8 | 3.3±0.3 |
| 45 | DAF-1/PKC, GSK-3 | CYQSARP**S**PEISHFG | 3.3±1.3 | 0 | 3.1±1.5 |
| 47 | DAF-18/SRC(kinase) | EEQAMEN**YT**NYGMIP | 0 | 0 | 3.3±0.9 |
| 50 | DAF-18/PKA | VQUIRRE**T**CPELHPED | 1.3±0.6 | 0 | 2.3±0.4 |
| 52 | DAF-2/ PKC, SGK-1 | MWREVEV**S**P**Y**EEAEK | 1.1±0.8 | 0 | 0 |
| 54 | *mus* AMPK/AMPK-1 | MSDGEFLR**T**SCGSPNY | 2.3±0.8 | 0 | 0 |
| 55 | *mus* ACC-1/PKA | NIPTLNRM**S**FASNLNH | 2.1±0.4 | 1.2±0.7 | 0 |
| 58 | *Hs* ACC-1/AMPK-2 | LALHIRS**S**W**S**GLH | 0 | 0 | 2.3±0.5 |
| 63 | *Hs* lipase/PKA, AMPK | IAEPMRR**S**V**S**EAA | 2.1±0.5 | 0 | 0 |
