# Supplementary material for: Positive Feedback between Transcriptional and Kinase Suppression in Nematodes with Extraordinary Longevity and Stress Resistance
Source: PLoS Genet. 2009 Apr 10;5(4):e1000452. doi: 10.1371/journal.pgen.1000452 (PMC2661368; doi:10.1371/journal.pgen.1000452)
Supplement: Table S2 — Gene expression data, quantified by real-time polymerase chain reaction, comparing three age-1 mutant groups to daf-2(e1370) mutants. Worms in the age-1(mg44) F2 group are sterile adults; others are post-gravid adults. Significance of difference from N2DRM (t-test): °, nominally significant at P<0.05; *, P<0.01; **, P<0.001; ***, P<1E−4; ****, P<1E−5, *****, P<1E−6. (0.05 MB DOC) [file pgen.1000452.s005.doc]

| **Pathway** | **Gene** | **Function / Notes .** | ***age-1 (mg44)*F1** | ***age-1* (*mg44)*F2** | ***age-1 (hx546)*** | ***daf-2 (e1370)*** |
| --- | --- | --- | --- | --- | --- | --- |
| **Insulin/IGF-1 Signaling (IIS) pathway** | | |  |  |  |  |
|  | ***ins-1*** | IIS antagonist | − | **10.0***** | **16.0*** | **8.0*** |
|  | ***ins-7*** | IIS agonist; RNAi extends life | 0.95 | **0.55º** | **5.3*** | 1.6 |
|  | ***age-1*** | PI3Kcs (class-I) | **0.07***** | **0.07******* | 1.6 | 1.5 |
|  | ***daf-18/pten*** | PIP_3_ phosphatase (opposes AGE-1) | **0.35º** | **0.08******* | 1.8 | 2.0 |
|  | ***sgk-1*** | serum/glucocorticoid-dep’t kinase | **0.18*** | **0.25*** | 1.0 | **2.5*** |
| **TGF-beta signaling**  ***daf-7*** | | TGF-β family member (agonist) | **7.0*** | **5.3**** | 2.6 | **4.9**** |
|  | ***daf-1*** | TGF-β family receptor, type I | **0.50º** | **0.22******* | **2.2º** | **2.5º** |
|  | ***daf-4*** | TGF-β family receptor, type II | 0.60 | **0.24***** | 1.5 | 1.1 |
|  | ***daf-3*** | co-SMAD transcription factor | **0.30***** | **0.30**** | **1.5º** | **1.9** |
| **AMPK/TOR pathway** | |  |  |  |  |  |
|  | ***aak-2*** | AMP-dep’t kinase 2 (activates DAF-16) | **0.60**** | **2.2**** | **1.7º** | **4.0*** |
|  |  |  |  |  |  |  |
|  | | | | | | |
|  |  |  |  | | | |
